# Heat‐killed *Lactobacillus murinus* confers neuroprotection against dopamine neuronal loss by targeting NLRP3 inflammasome

**DOI:** 10.1002/btm2.10455

**Published:** 2022-11-23

**Authors:** Hong‐Xia Fan, Shuo Sheng, Dai‐Di Li, Jing‐Jie Li, Guo‐Qing Wang, Feng Zhang

**Affiliations:** ^1^ Key Laboratory of Basic Pharmacology of Ministry of Education and Joint International Research Laboratory of Ethnomedicine of Ministry of Education and Key Laboratory of Basic Pharmacology of Guizhou Province and Laboratory Animal Center Zunyi Medical University Zunyi Guizhou China

**Keywords:** *Lactobacillus murinus*, microglia, neuroprotection, NLRP3 inflammasome, Parkinson's disease

## Abstract

The intestinal flora has become very active in studies related to Parkinson's disease (PD) in recent years. The microbe‐gut‐brain axis is closely related to the maintenance of brain homeostasis as well as PD pathogenesis. Alterations in gut bacteria can contribute to neuroinflammation and dopamine (DA) neurodegeneration. *Lactobacillus murinus*, a gram‐positive bacterium, is a commensal gut bacteria present in the mammalian gut and considered as a potential probiotic due to its beneficial effects, including anti‐inflammatory and antibacterial actions. In this study, the effects of live *L. murinus* and heat‐killed *L. murinus* on DA neuronal damage in rats and the underlying mechanisms were investigated. Data showed that heat‐killed *L. murinus* ameliorated 6‐hydroxydopamine‐induced motor dysfunctions and loss of substantia nigra DA neurons, while no protection was shown in live *L. murinus* treatment. At the same time, heat‐killed *L. murinus* reduced the activation of NLRP3 inflammasome in microglia and the secretion of pro‐inflammatory factors, thus inhibiting the development of neuroinflammation. Furthermore, heat‐killed *L. murinus* failed to display its original neuroprotective properties in NLRP3 inflammasome knockout mice. Together, heat‐killed *L. murinus* conferred neuroprotection against DA neuronal loss via the inhibition of microglial NLRP3 inflammasome activation. These findings provide a promising potential for future applications of *L. murinus*, and also beneficial strategy for PD treatment.

## 1 INTRODUCTION

Parkinson's disease (PD) is one of most common neurodegenerative diseases affecting about 1–5% of the world's people over the age of 60 years old.^1^, ^2^ Degeneration and loss of dopamine (DA) neurons in substantia nigra was the main pathological feature of PD patients. With the progression of the disease, motor symptoms (tremor, stiffness, bradykinesia) and non‐motor symptoms (cognitive decline, constipation, and sleep disturbance) superimpose.^3^, ^4^ Although the exact pathogenesis of PD remains to be elucidated, studies on a large number of PD patients, animal models and cell experiments have shown that neuroinflammation plays an important role in the occurrence and development of PD.^5^, ^6^, ^7^

Microglia are important innate immune cells in the central nervous system (CNS). When inflammation or other pathogenic factors attack the nervous system of the brain, microglia respond rapidly, and the activated microglia change their morphology and release various inflammatory mediators, such as IL‐1β, IL‐18, and TNF‐α. Studies have shown that these cytokines are involved in mediating neuroinflammation and acute or chronic neurodegeneration in CNS pathology. IL‐1β, normally expressed at low levels, activates microglia when secreted in large quantities.^8^, ^9^ Moreover, IL‐18 can induce interferon‐γ through natural killer cells, guide autoreactive T cells, and promote autoimmune neurodegeneration in CNS.^10^ TNF‐α in physiological state participates in homeostasis regulation and synaptic plasticity. However, massively TNF‐α production induces neuronal damage through excessive release of glutamate and inhibition of astrocytic glutamate reuptake, resulting in neuronal excitotoxicity and death.^9^ The secretion of these cytokines is closely related to the NOD‐like receptor protein 3 (NLR family pyrin domain containing 3, NLRP3) inflammasome in microglia. Current evidence indicates that the NLRP3 inflammasome complex is upregulated in microglia of the substantia nigra in PD patients and PD animal models.^11^, ^12^ In addition, NLRP3 sensor, apoptosis‐associated card‐containing speck‐like protein (ASC) and caspase‐1 form the inflammatory complex,^13^, ^14^ resulting in caspase‐1‐dependent activation and secretion of the pro‐inflammatory cytokines, IL‐1β, and IL‐18.^15^ These pro‐inflammatory factors further stimulate the activation of microglia, leading to persistent and slow neuroinflammation in the brain. Inhibition of the NLRP3 inflammasome signaling activation could alleviate the progression of PD.^16^, ^17^ Therefore, the NLRP3 inflammasome in microglia might be an effective target for PD therapy.

Research on the intestinal flora in relation to neurodegenerative diseases, such as PD, has become very active in recent years. The microbe‐gut‐brain axis is closely related to the maintenance of brain homeostasis as well as PD pathogenesis.^18^, ^19^ Alterations in gut bacteria can contribute to neuroinflammation and DA neurodegeneration by promoting enteric and peripheral neurogenic/inflammatory responses.^20^ In particular, NLRP3 inflammasome actively participates in or shapes peripheral and central immune/inflammatory responses in CNS diseases. NLRP3 inflammasome activation affected gut microbial composition, and gut microbial dysbiosis could also lead to overactivation of the NLRP3 inflammasome pathway.^21^, ^22^, ^23^ Clinical studies have shown that the gut microbiome was altered in PD patients, in addition to the activation of the NLRP3 inflammasome in the gut, periphery, and brain, which may suggest that microbes, NLRP3 inflammasome and PD interact with each other.^24^ Today, a growing body of studies confirms the beneficial effects of probiotics and heat‐killed microorganisms on the host. Probiotics or inactivated microorganisms can play an immunomodulatory role in modulating NLRP3 inflammasome activity.^25^, ^26^ As COVID‐19 swept the world in the past 2 years, probiotics have been discussed as a potential treatment for COVID‐19.^27^, ^28^ In addition, inactivated microorganisms have been considered as candidate immunomodulators for COVID‐19.^29^ Thus, probiotics or inactivated microbial preparations have been proposed as therapeutic or prophylactic preparations. Collectively, the development of probiotics or inactivated microorganisms for the treatment of PD might have promising applications.

In this study, high‐throughput sequencing technology was first performed to detect the changes of intestinal flora in PD rat model induced by 6‐hydroxydopamine (6‐OHDA), lipopolysaccharide (LPS), and rotenone (ROT). A differentially altered potential probiotic, *Lactobacillus murinus (L. m)*, was found in the feces of the 6‐OHDA model. *L. m* is a gram‐positive bacterium belonging to the Firmicutes and Lactobacillus families. Recent studies have shown that *L. m* not only reduced the transport of bacterial products and markers of systemic inflammation in mice, but also improved neurobehavioral and microglial dysfunction in offspring induced by antibiotic‐based models of maternal dysbiosis.^30^, ^31^ This suggests that *L. m* could have potential neuroprotective properties. However, the underlying mechanism is unclear. In the present study, 6‐OHDA‐induced rat DA neuronal loss was applied to explore the neuroprotective effects of live *L. m* and heat‐killed *L. m* (H‐k *L. m*) and the possible mechanisms. These findings would provide a promising potential for future applications of *L. m* and also beneficial strategy for PD treatment.

## 2 MATERIALS AND METHODS

### 2.1 Reagents

ROT (Catalog No. R8875), LPS (Catalog No. L6143), and 6‐OHDA (Catalog No. H4381) were obtained from Sigma Chemical (St. Louis, MO, USA). Lysis buffer and the enhanced chemiluminescence (ECL) reagent were purchased from Beyotime Institute of Biotechnology (Shanghai, China). Anti‐tyrosine hydroxylase (TH) (Catalog No. 25859‐1‐AP; rabbit polyclonal), IL‐1β (Catalog No. 16806‐1‐AP; rabbit polyclonal), TNF‐α (Catalog No. 17590‐1‐AP; rabbit polyclonal), NLRP3 (Catalog No. 19771‐1‐AP; rabbit polyclonal), caspase‐1/p20/p10 (Catalog No. 22915‐1‐AP; rabbit polyclonal), and GAPDH (Catalog No. 10494‐1‐AP; rabbit polyclonal) antibodies were bought from Proteintech Group (Chicago, IL, USA). Anti‐ionized calcium binding adaptor molecule‐1 (IBA‐1; Catalog No. ab178847; rabbit monoclonal), Alexa Fluor 594 (Catalog No. ab 150,080, goat anti‐rabbit) and Alexa Fluor 488 (Catalog No. ab 150077, goat anti‐rabbit) antibodies were bought from Abcam (Cambridge, MA, USA). Anti‐ASC (Catalog No. abs155599; rabbit polyclonal), and IL‐18 (Catalog No. abs120003; rabbit polyclonal) antibodies were obtained from Absin (Pudong New District, Shanghai, China). Anti‐NLRP3 (Catalog No. BA3677; Goat Polyclonal) antibody was bought from Boster (Wuhan, China).

### 2.2 Animals and treatment

Male Sprague–Dawley rats (180–220 g) were purchased from Beijing HFK Bioscience Co., Ltd and NLRP3 knockout mice were from Jiangsu Aniphe Biolaboratory Inc. Animals were raised in an environment where the temperature was maintained at 19–24°C and the humidity was kept in 40–70% with free access to food and water. Mice were genetically identified before being used in experiments. Rats were used for adaptive feeding for 7 days before experiment. All animal experiments were conducted in accordance with the Guidelines for Animal Protection and Welfare in China and approved by the Animal Care and Use Committee of Zunyi Medical University (Zunyi, China). Rats/mice were injected with 6‐OHDA (15 μg for rats^32^; 4 μg for mice,^33^ 0.2% ascorbic acid in saline) or LPS (5 μg, in saline) solution into the unilateral substantia nigra in midbrain. Bregma was the origin of the coordinates. Coordinates of rats were 5.2 mm posterior to the bregma, 2.2 mm lateral to the midline, and 8.1 mm ventral to the surface of the skull. Mouse coordinates were 2.2 mm posterior to the bregma, 1.4 mm lateral to the midline, and 4.7 mm ventral to the surface of the skull. ROT (0.5 mg/kg/d) was subcutaneously injected in rats six times a week for consecutive 4 weeks.

### 2.3 Culture and treatment of *L. m*

*L. m* (CICC23140) was activated and passaged to restore viability. *L. m* was inoculated in methane‐rich saline medium and cultured at 37°C for 8 h. The OD value of the bacterial solution was measured with an UV spectrophotometer at 600 nm. When OD value was 0.8, the concentration of bacterial solution was 2.8 × 10^8^ cfu/ml. Then, the bacteria were centrifuged for 20 min at 4°C and the culture medium was discarded. Bacteria were mixed with water to obtain live *L. m*, or then inactivated at 121°C for 20 min to obtain H‐k *L. m*. Live *L. m* or H‐k *L. m* was administrated in rats/mice by intragastric gavage daily for 3 weeks previous 6‐OHDA injection (detail described in Animals and Treatment section) and followed for further 2 weeks.

### 2.4 16S rRNA sequencing

After the modeling period, the rat feces were collected in sterile centrifuge tubes and stored at −80°C for analysis. Sample DNA was extracted by cetyltrimethyl ammonium bromide method. Appropriate amount of sample DNA was put into the centrifuge tube and diluted to 1 ng/μl with sterile water. Using the diluted genomic DNA as the template, specific primers, such as Barcode, Phusion high‐fidelity PCR Master Mixwith New England Biolabs GC Buffer were used. Based on the selection of sequencing regions, PCR was performed with high fidelity enzyme. PCR products were detected by 2% agarose gel electrophoresis. The library was constructed using TruSeqDNA PCR‐free sample preparation kit library construction kit, and the constructed library was quantified by qubits and Q‐PCR. After the library was qualified, NovaSeq 6000 was used for sequencing on the machine.

### 2.5 Metagenomics

DNA concentrations were determined using the Qubit dsDNA assay kit after DNA extraction from fecal samples, with OD values between 1.8 and 2.0 and DNA content above 1 μg to construct libraries. DNA was randomly interrupted into fragments of approximately 350 bp in length using a Covaris ultrasonic disruption instrument, and PCR products were purified by end repair, A‐tail addition, and sequencing adapters (AMPure XP system). Initial quantitation was performed using Qubit 2.0, diluting the library to 2 ng/μl, followed by detection of inserts into the library using Agilent 2100, and accurate quantitation of the effective concentration of the library (effective concentration of the library >3 nM) was performed using Q‐PCR method after inserts were as expected. The index‐encoded samples were clustered on a cBot cluster generation system. After cluster generation, Illumina PE150 sequencing was performed and paired‐end reads were generated.

### 2.6 Rotarod test

Rotarod test is a common test applied to assess animal behavior dysfunctions. In detail, animals were placed on the rotating shaft rod of the fatigue meter, and the corresponding acceleration parameters were selected for the experiment (4.0–40 rpm/0–30 rpm) until animals fell off the rotating rod. The exercise ability of animals was measured according to the setting program of fatigue instrument, and the time animals stayed on rod was recorded. Each animal was tested for three times with an interval of more than 30 min. The mean value was used for statistical analysis.

### 2.7 Forepaw adjusting steps test

Forepaw adjusting steps test was conducted 1 d after last *L. m* treatment. In detail, the posterior two legs and one forepaw of rat were fixed so that the rat body weight was supported by only one forelimb (the posterior two legs were suspended by lifting the tail of mouse and the body was supported by two forepaws). Rats were moved 90 cm forward at a constant speed within 10 s (mice were moved 90 cm backward at a constant speed within 4 s). The number of adjusted steps of the rat/mouse forelimb was recorded, and the number of adjusted steps of the other forelimb was measured by the rat simultaneous method. The test was repeated three times and results were represented by (number of moving steps on the injured side/number of moving steps on the injured side) × 100%.

### 2.8 Western blotting

Animals were sacrificed after deep anesthesia and brains were quickly removed and midbrain tissues were isolated on ice and cryopreserved at −80°C. The tissue was homogenized by adding lysis buffer (containing protease inhibitors), and the supernatant collected by centrifugation after lysis on ice was the proprotein solution. The stock solution was diluted 50‐fold and quantified using a BCA protein quantification kit. After adding protein stock solution, PBS and loading buffer to centrifuge tubes, the samples used for immunoblotting were denatured at 100°C. Equal amount of sample was loaded on sodium dodecyl sulfate polyacrylamide electrophoresis gel (10 or 12.5%), electrophoresis parameters were first set as 70 V, and then pressurized to 110 V after 50 min. According to the target molecular weight, the protein was transferred to PVDF membrane and sealed with 5% skim milk for 2 h. The following primary antibodies were incubated at 4°C for 16 h: rabbit anti‐TH (the marker of DA neurons, 1:2000; Proteintech), rabbit anti‐IL‐1β (1:1000; Proteintech), rabbit anti‐TNF‐α (1:1000; Proteintech), rabbit anti‐IBA‐1 (the marker of microglia, 1:1000; Abcam), rabbit anti‐caspase‐1 (1:1000; Proteintech), rabbit anti‐NLRP3 (1:1000; Boster Biological), rabbit anti‐ASC (1:1000; Absin), rabbit anti‐IL‐18 (1:1000; Absin), and anti‐GAPDH (1:5000; Proteintech) antibodies. The HRP‐conjugated anti‐rabbit secondary antibody (1:5000; Proteintech) was combined with the primary antibody for 40 min at room temperature. Chemiluminescence reagent (ECL kit) was used to display the blotting, and Image Lab 6.0 software was used to quantify the blotting.

### 2.9 Immunohistochemical and immunofluorescence staining

Paraffin sections were deparaffinized sequentially in xylene and different concentrations of ethanol. The sections were immersed in citrate and subject to moderate to high temperature repair. After cooling, they were incubated with 10% goat serum for 30 min at 37°C. *For immunohistochemical staining*, an anti‐TH (1:500, Proteintech) antibody was added after discarding the goat serum and incubated overnight at 4°C. After washing by PBS, biotinylated secondary antibody working solution was dropped and reacted at 37°C for 20 min. After washing with PBS again, the working solution of horseradish conjugated streptavidin was dropped and reacted at 37°C for 20 min. The sections were stained with Diaminobenzidine (DAB) and then photographed. *For immunofluorescence staining*, the following primary antibodies were incubated at 4°C overnight: anti‐TH (1:200; Proteintech), IBA‐1 (1:250; Abcam), and NLRP3 (1:200; Proteintech) antibodies, and then washed with PBS and incubated at 37°C for 60 min in the dark with secondary antibodies: anti‐rabbit IgG Alexa Flour 488 (1:500; Abcam) or anti‐rabbit IgG Alexa Flour 594 (1:500; Abcam) antibodies. After PBS rinsing, the slides were mounted with glycerol. All the results were analyzed and processed with ImageJ software after photography.

### 2.10 Statistical analysis

Data were showed as mean ± standard error of the mean (*SEM*) using GraphPad Prism software. Statistical significance between two groups was analyzed by unpaired *t*‐test. Significance among multiple groups was analyzed by one‐way analysis of variance (ANOVA) followed by Bonferroni's post hoc test. Values of *p* < 0.05 were considered statistically significant. In addition, the correlation between the degree of TH injury and the level of IBA‐1 activation as well as the level of IBA‐1 and NLRP3 activation was tested by Pearson correlation analysis.

## 3 RESULTS

### 3.1 Changes of gut bacteria in PD rat models

**FIGURE 1:**
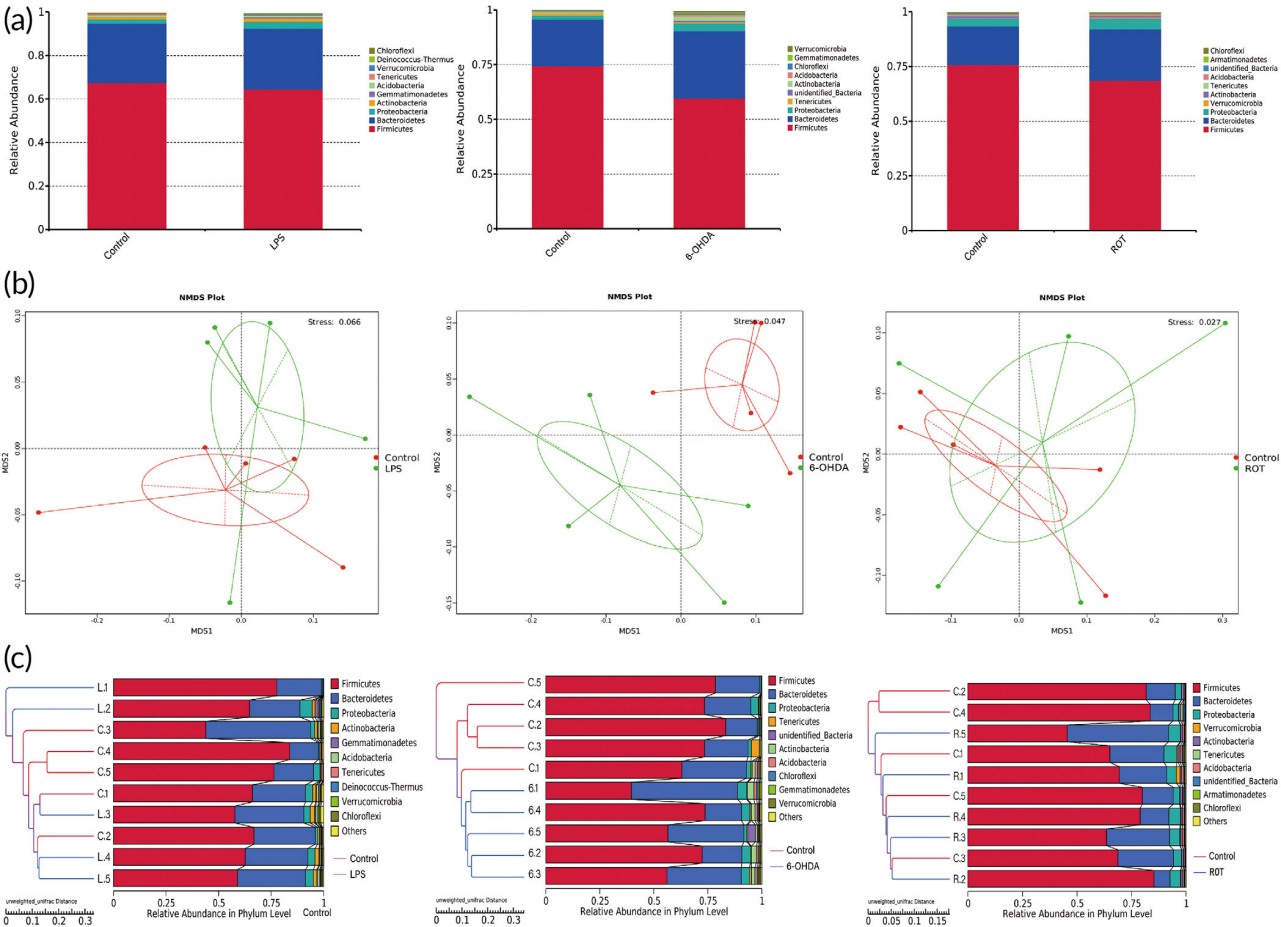
Changes of gut bacteria in Parkinson's disease (PD) rat models. Species relative abundance of microorganisms in feces was detected by 16S rRNA sequencing technique (a, phylum level), nonmetric multidimensional scaling (NMDS) analysis (b) and unweighted pair‐group method with arithmetic mean (UPGMA) analysis (c) after application of lipopolysaccharide (LPS), 6‐hydroxydopamine (6‐OHDA), and rotenone (ROT). Stress <0.2 indicated that the NMDS analysis was reliable. Data were represented from five rats in each group.

Three common rat PD models were prepared by midbrain injection of LPS or 6‐OHDA as well as subcutaneous injection of ROT in the back of the neck. 16S rRNA sequencing technology was used to detect the changes of gut bacteria in these 3 models. 16S rRNA sequencing technology was based on Illumina Nova sequencing platform sequencing to construct PCR‐free libraries, followed by paired‐end sequencing, clustering sequences into Operational Taxonomic Units (OTUs) with 97% consistency, and then species annotation of OTUs sequences with the Silva132 database. As a result, Figure 1a presented the species relative abundances of gut bacteria in 3 PD rat models, which were made based on the results of species annotation, showing the top 10 abundances of bacteria at phylum level. Firmicutes and Bacteroidetes were seen to be the most predominant phyla among rat gut bacteria, accounting for the predominant relative abundance in all samples. Although the sequencing results indicated that the gut bacteria of three PD models exhibited a decreasing trend of Firmicutes and an increasing trend of Bacteroidetes, these changes were most obvious in 6‐OHDA model than LPS or ROT models.

Next, to explore the characteristic changes of gut bacteria in PD rat models, a comparative analysis of microbial community composition was performed based on species annotation results and abundance information of OTUs from all samples. As shown in Figure 1b, nonmetric multidimensional scaling (NMDS) statistical analysis was performed for each of these three PD models. NMDS was used to investigate the differences of samples and groups. The results of NMDS analysis based on OTUs levels indicated that 6‐OHDA had gradually formed a microbial community different from the control group, while LPS and ROT did not form a significantly independent community. Furthermore, Figure 1c was the result of the unweighted pair‐group method with arithmetic mean (UPGMA) cluster tree analysis. According to the OTU analysis results of each sample, we used different algorithms to measure the dissimilarity coefficient between the two samples. The ordinate value corresponding to the branch node was the distance between the two samples/clusters. If the value was lower, the diversity difference between the two samples was smaller. Therefore, the similarity relationship of gut microbiota composition between two groups could be judged by UPGMA clustering tree. Consistent with the results of NMDS analysis, the UPGMA clustering results demonstrated that the control group corresponding to 6‐OHDA group showed clusters respectively, indicating that the species composition and structure of the groups were similar. However, there was no significant difference of clustering between ROT or LPS and control groups.

**FIGURE 2:**
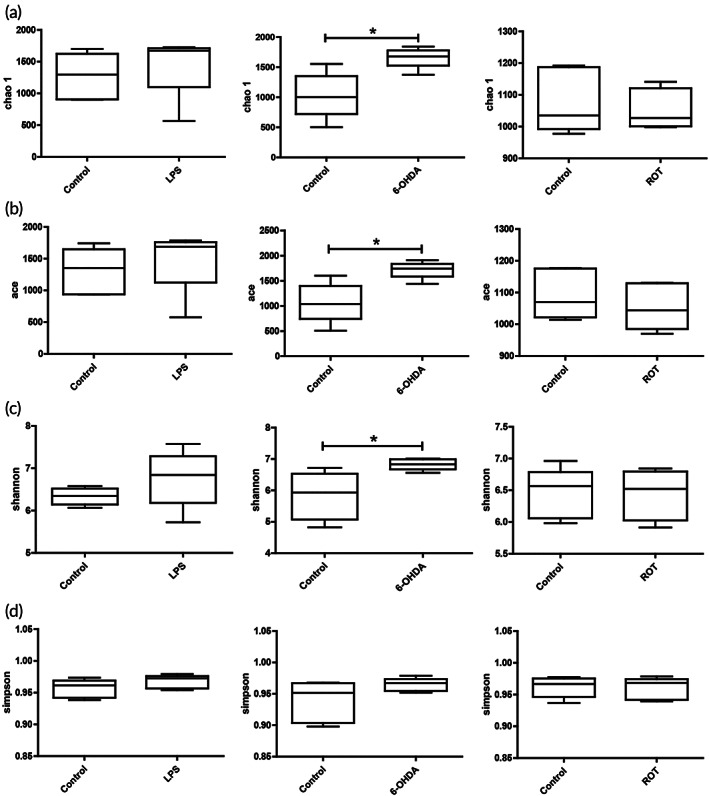
Intestinal bacterial diversity was investigated in various Parkinson's disease (PD) rat models. The diversity changes of intestinal bacteria in 6‐hydroxydopamine (6‐OHDA), lipopolysaccharide (LPS), and rotenone (ROT)‐induced PD rat models were analyzed by chao 1 index (a), ace index (b), shannon index (c), and simpson index (d). Data were expressed as mean ± *SEM* from five rats. *p < 0.05 compared with control group.

To further determine whether the changes in the diversity of intestinal bacteria were statistically significant, α‐diversity statistical analysis was performed. Chao 1 index and ace index were used to assess species richness (OTU number). Shannon index and simpson index were used to comprehensively evaluate species richness and evenness. The results showed that chao 1 index and ace index were significantly increased in the 6‐OHDA group compared with the control group (Figure 2a,b). In addition, shannon index increased significantly in the 6‐OHDA group compared with the control group, while simpson index showed no significant difference (Figure 2c,d). In addition, there were no statistically significant changes in the four diversity indexes in LPS and ROT groups. These results suggest that 6‐OHDA treatment can significantly change the abundance and diversity of intestinal flora in rats.

### 3.2 *L. m* changed apparently in the 6‐OHDA model

**FIGURE 3:**
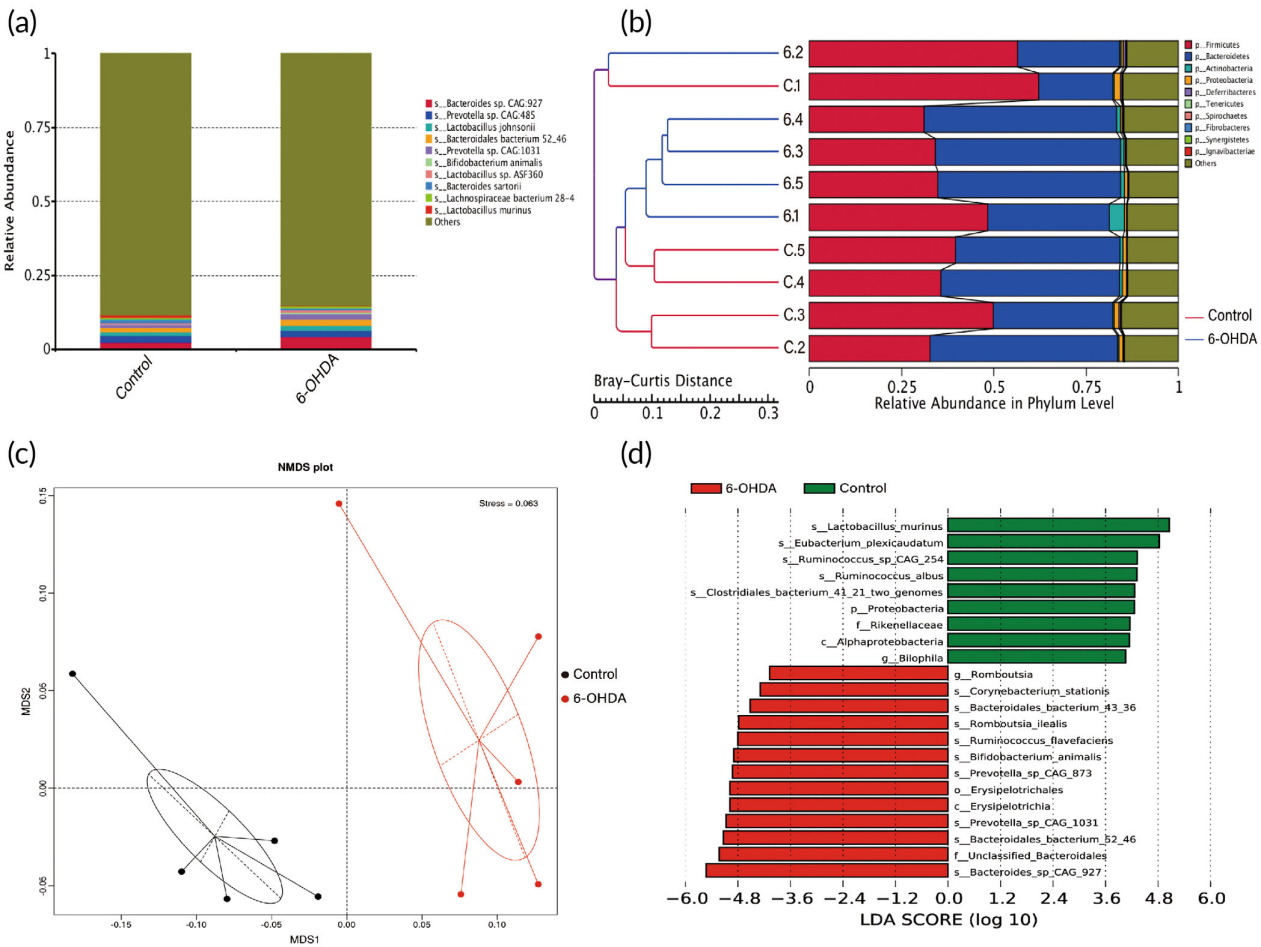
*L. m* changed apparently in the 6‐hydroxydopamine (6‐OHDA) model. Species relative abundance (species level) of gut bacteria in 6‐OHDA model was explored by metagenome (a) and unweighted pair‐group method with arithmetic mean (UPGMA) analysis (b), nonmetric multidimensional scaling (NMDS) analysis (c), and LDA Effect Size analysis (d). The threshold for the logarithmic LDA score was 4.0 and *p* < 0.05 for the factorial Kruskal–Wallis test.

The above results showed that compared with LPS and ROT models, gut microbiota in 6‐OHDA model changed obviously. Next, metagenomic sequencing was performed on 6‐OHDA model. As shown in Figure 3a, changes in the relative abundance of the top 10 species at the species level in 6‐OHDA group compared to control group were exhibited. Furtherly, UPGMA clustering analysis showed good clustering (Figure 3b). Also, results of NMDS analysis matched those of 16S rRNA analysis. The 6‐OHDA group was different from the control group, and these two groups formed independent microbial communities (Figure 3c). Using LDA Effect Size analysis, it could be seen from the LDA value distribution histogram that a variety of bacteria was present with significant differences between these two groups. Collectively, *L. m*, the bacterium with the most significant changes, was selected for potential as a probiotic in the following studies (Figure 3d).

### 3.3 Effects of *L. m* on 6‐OHDA‐induced DA neurotoxicity

**FIGURE 4:**
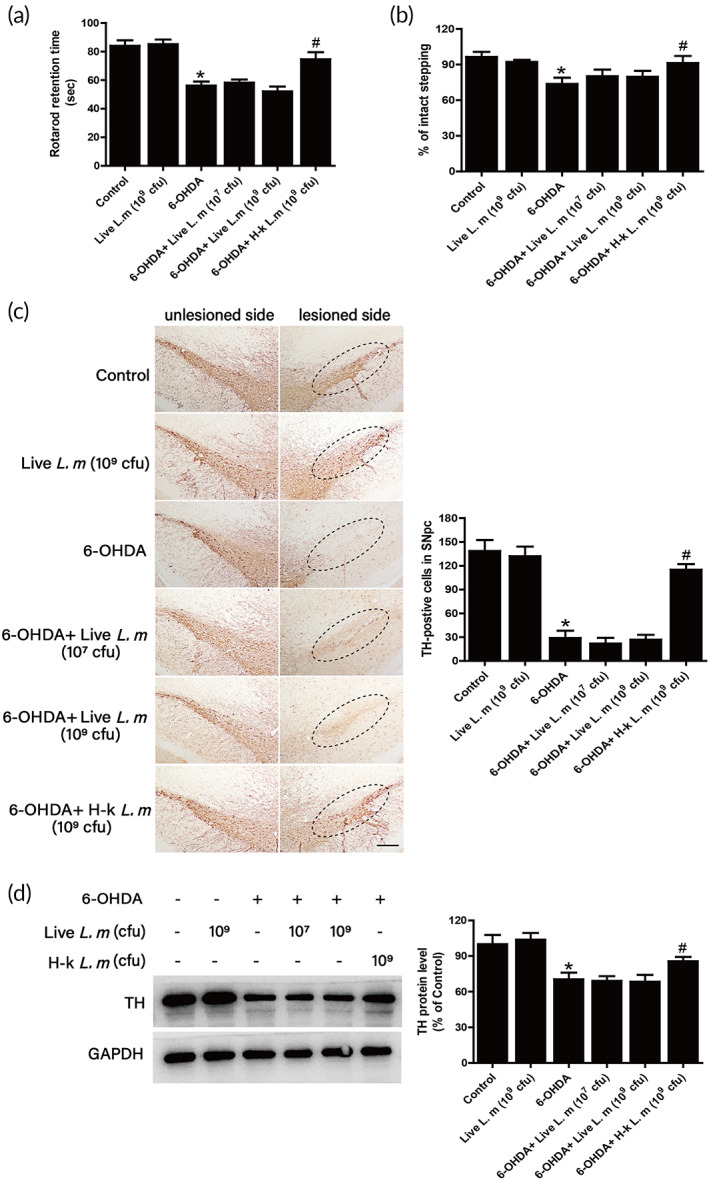
Effects of *L. m* on 6‐hydroxydopamine (6‐OHDA)‐induced dopamine (DA) neurotoxicity. Rat behavioral changes were assessed by the rotarod test (a) and the stepping test (b) 1 day after the last administration of live *L. m* or H‐k *L. m*. Brain tissues were collected after rats were sacrificed. DA neurons were labeled by immunohistochemical staining with an anti‐tyrosine hydroxylase (TH) antibody (c). The “ellipses” indicated areas of substantia nigra. The loss of DA neurons in the substantia nigra was analyzed by counting the number of TH‐positive neurons. Scale = 100 μm. TH protein expression was determined by western blotting (d). Data were expressed as mean ± *SEM* from five to eight rats. *p < 0.05 compared with control group; ^#^ *p* < 0.05 compared with 6‐OHDA group.

After administration of live *L. m* and H‐k *L. m* for five consecutive weeks, it was seen from the results of two behavioral tests in Figure 4a,b. Compared with the control group, a longer time rats stayed on the rod was recorded and the number of adjusted steps of the forelimb on the injured side was reduced in 6‐OHDA‐induced PD rat model. Compared with 6‐OHDA group, H‐k *L. m* rather than live *L. m* attenuate rat motor dysfunctions. To further determine the effects of *L. m* on 6‐OHDA‐induced DA neuronal loss in rats, the quantification of TH‐positive DA neurons and the expression of TH protein in substantia nigra were investigated. As shown in Figure 4c, live *L. m* had no protective effects on DA neuronal damage induced by 6‐OHDA, whereas H‐k *L. m* reduced 6‐OHDA‐induced DA neuronal loss. Similar results were shown in TH protein level measurement (Figure 4d).

**FIGURE 5:**
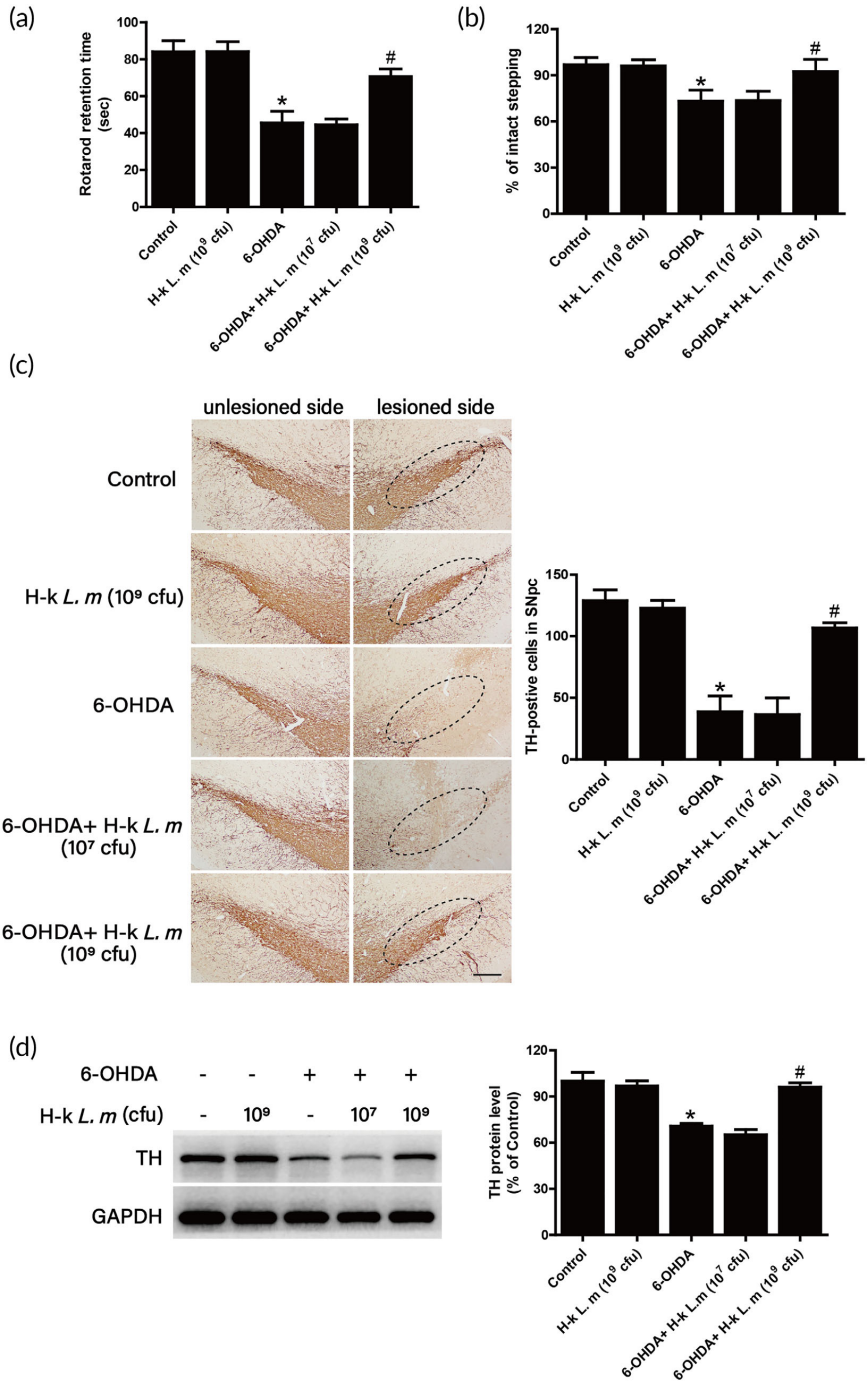
H‐k *L. m* exerted neuroprotection against 6‐hydroxydopamine (6‐OHDA)‐induced dopamine (DA) neurotoxicity. Behavioral changes of rats were assessed by the rotarod test (a) and the stepping test (b) 1 day after the last administration of H‐k *L. m*. DA neurons were labeled by immunohistochemical staining (c). The “ellipses” indicated the areas of substantia nigra. The loss of DA neurons in the substantia nigra was analyzed by counting the number of tyrosine hydroxylase (TH)‐positive neurons. Scale = 100 μm. TH protein expression was determined by western blotting (d). Data were presented as mean ± *SEM* from five to nine rats. *p < 0.05 compared with control group; ^#^ *p* < 0.05 compared with 6‐OHDA group.

Since H‐k *L. m* presented DA neuroprotection, the effects of different doses of H‐k *L. m* on DA neuronal damage were next investigated. Behavioral tests analysis showed that H‐k *L. m* could increase the exercise time rat stayed on the rod and the number of forelimb adjustment steps on the injured side of rats compared with the 6‐OHDA group (Figure 5a,b). Also, immunohistochemical staining results indicated that H‐k *L. m* effectively antagonized the loss of DA neurons induced by 6‐OHDA (Figure 5c). Moreover, the result from TH protein level measurement (Figure 5d) was consistent with DA neuronal quantification. These results suggested that H‐k *L. m* produced neuroprotection against 6‐OHDA‐induced DA neurotoxicity.

### 3.4 H‐k *L. m* attenuated 6‐OHDA‐induced microglial activation and inflammatory factors release

**FIGURE 6:**
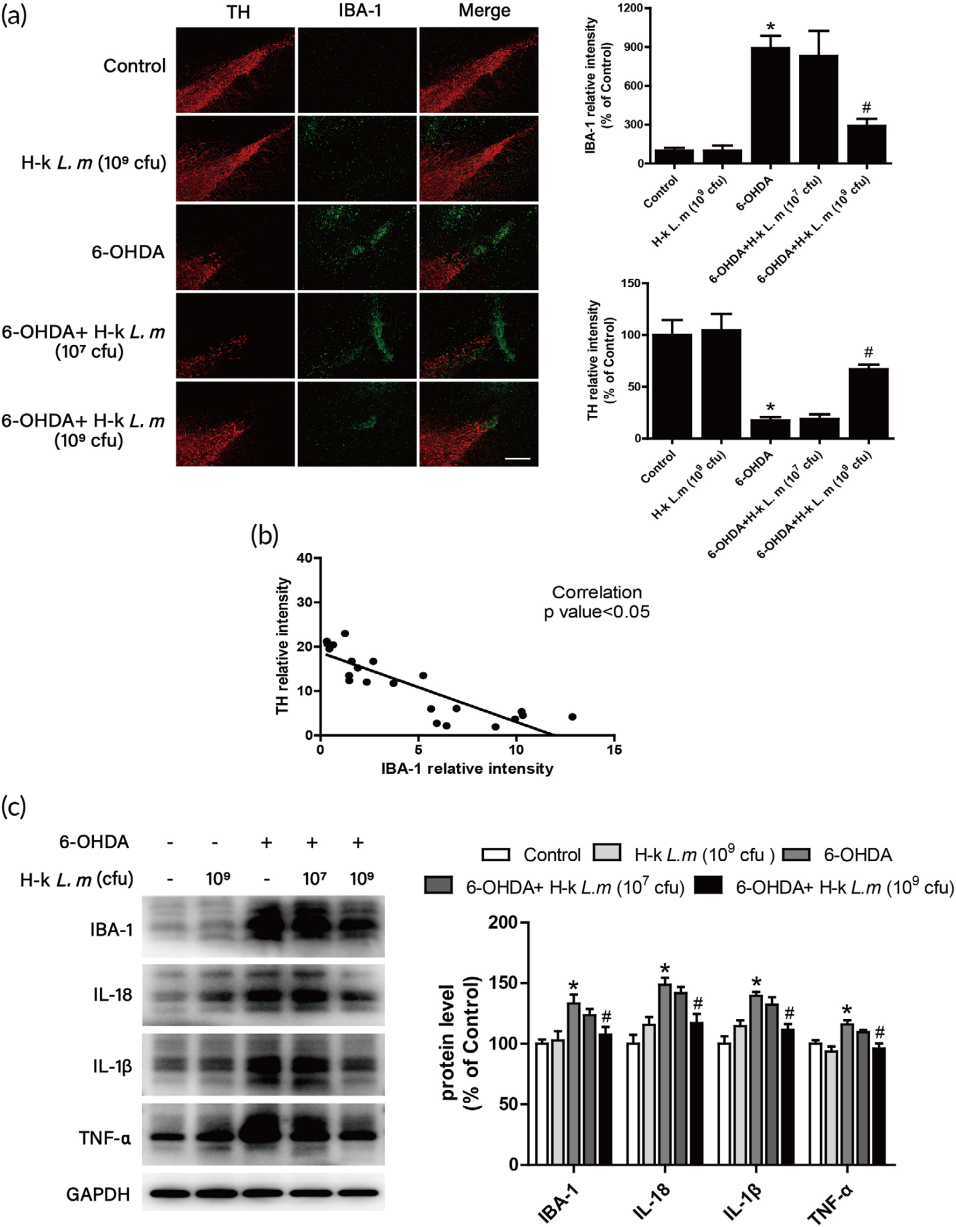
H‐k *L. m* attenuated 6‐hydroxydopamine (6‐OHDA)‐induced microglial activation and release of inflammatory factors. Rat brains were collected and sectioned. Double immunofluorescence staining with anti‐IBA‐1 (green) and tyrosine hydroxylase (TH) (red) antibodies was performed (a). The fluorescence intensity of staining was quantified. Scale = 100 μm. Correlation analysis of TH and IBA‐1 expressions was determined (b). The protein expression levels of IBA‐1, IL‐18, IL‐1β, and TNF‐α in rat midbrain were determined by western blotting (c). Data were presented as mean ± *SEM* from five rats. *p < 0.05 compared with control group; ^*#*^ *p* < 0.05 compared with 6‐OHDA group.

Next, the effects of H‐k *L. m* on microglia‐mediated neuroinflammation were investigated. First, the expressions of TH (DA neuron marker) and IBA‐1 (microglia marker) in rat substantia nigra were detected by immunofluorescence staining. As shown in Figure 6a, compared with control group, 6‐OHDA reduced TH (red) protein level and increased IBA‐1 (green) protein level. After H‐k *L. m* treatment, H‐k *L. m* reversed 6‐OHDA‐induced changes of TH and IBA‐1. Then, the correlation between TH and IBA‐1 protein level was further analyzed. As shown in Figure 6b, there was a strong negative correlation between DA neuronal damage and microglia activation in the substantia nigra. Second, the expression of IBA‐1 protein was detected (Figure 6c). Consistent with the results of immunofluorescence staining, H‐k *L. m* reduced 6‐OHDA‐induced high protein expression of IBA‐1. At the same time, H‐k *L. m* also inhibited the protein expressions of inflammatory factors, such as TNF‐α, IL‐1β, and IL‐18, induced by 6‐OHDA (Figure 6c).

### 3.5 H‐k *L. m* inhibited NLRP3 inflammasome activation in microglia

**FIGURE 7:**
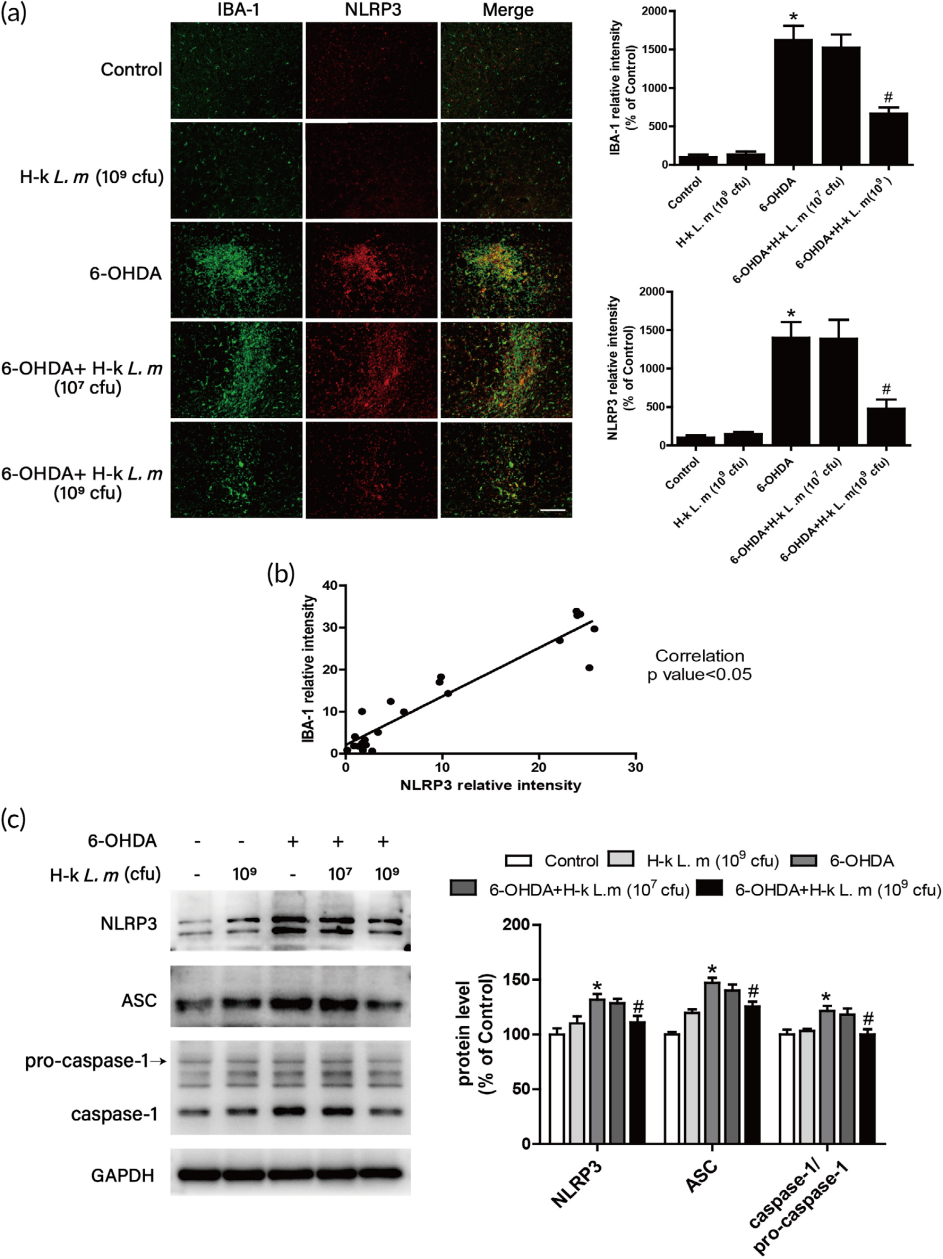
H‐k *L. m* inhibited NLRP3 inflammasome activation in microglia. Staining with anti‐NLRP3 (red) and IBA‐1 (green) antibodies was performed by double immunofluorescence (a). The fluorescence intensity of staining was quantified to assess NLRP3 and IBA‐1 levels. Scale = 50 μm. Correlation analysis between NLRP3 and IBA‐1 expression was performed (b). The protein levels of NLRP3, ASC, pro‐caspase‐1, and caspase‐1 in rat midbrain were determined by western blotting (c). Data were presented as mean ± *SEM* from five rats. *p < 0.05 compared with control group; ^*#*^ *p* < 0.05 compared with 6‐hydroxydopamine (6‐OHDA) group.

It was well known that the activation of microglia was closely related to the neuroinflammatory response.^34^ Here, the association between microglia and the NLRP3 inflammasome in the substantia nigra could be clearly seen by immunofluorescence co‐localization. As shown in Figure 7a, compared with control group, the NLRP3 inflammasome (red) was obviously activated in the areas where microglia (green) were activated in the 6‐OHDA group. After H‐k *L. m* treatment, the activation of microglia and the NLRP3 inflammasome was inhibited. The correlation analysis between IBA‐1 and NLRP3 showed that the activation of microglia was positively correlated with the expression of NLRP3 inflammasome (Figure 7b). In addition, H‐k *L. m* was also able to suppress 6‐OHDA‐induced activation of the NLRP3 inflammasome signaling, including NLRP3, ASC, and caspase‐1 (Figure 7c).

### 3.6 H‐k *L. m* exerted DA neuroprotection by inhibiting microglial NLRP3 inflammasome activation

**FIGURE 8:**
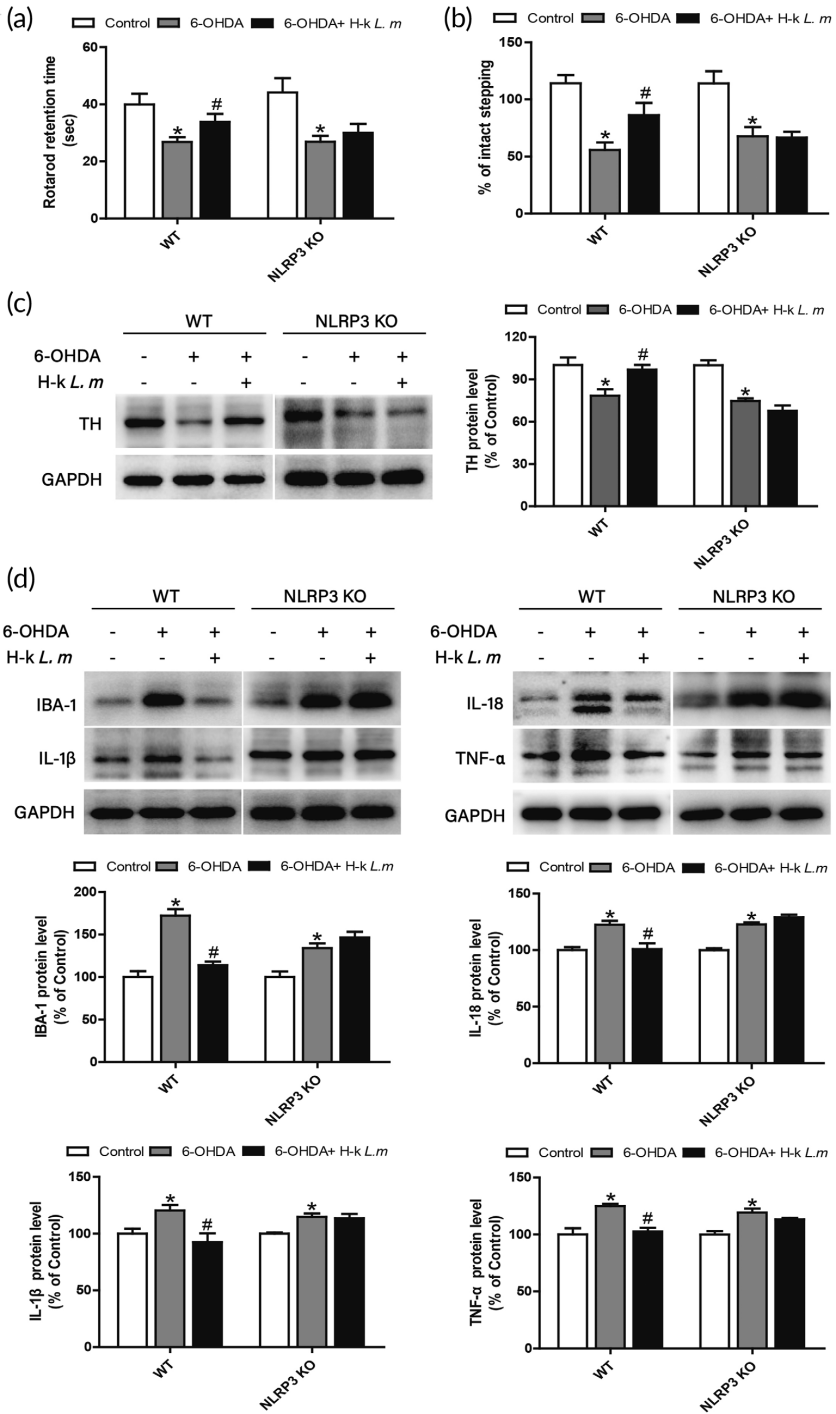
H‐k *L. m* exerted dopamine (DA) neuroprotection by inhibiting microglial NLRP3 inflammasome activation. H‐k *L. m* (1.4 × 10^9^ cfu) was administered in wild‐type (WT) and NLRP3 knockout (KO) mice. Mouse behavior dysfunctions were assessed by rotarod (a) and stepping assays (b). DA neurotoxicity was assessed by tyrosine hydroxylase (TH) protein expression detection (c). Microglia activation and inflammatory mediators levels were determined by IBA‐1, IL‐18, IL‐1β, and TNF‐α protein expressions measurement (d). Data were presented as mean ± *SEM* from five to nine rats. *p < 0.05 compared with control group; ^*#*^ *p* < 0.05 compared with 6‐hydroxydopamine (6‐OHDA) group.

To further explore whether H‐k *L. m‐*mediated DA neuroprotection was related to the inhibition of NLRP3 inflammasome activation, NLRP3 knockout mice was applied. As shown in Figure 8a,b, compared with 6‐OHDA group, H‐k *L. m* increased the rod exercise time and the number of steps adjusted in the forelimb of the injured side of wild‐type mice. However, H‐k *L. m* did not improve behavior dysfunctions in NLRP3 knockout mice. Subsequently, the protein expression of TH was detected. As shown in Figure 8c, H‐k *L. m* attenuated 6‐OHDA‐induced DA neuron damage in wild‐type mice, whereas no DA neuroprotection was discerned in NLRP3 knockout mice. In addition, H‐k *L. m* could inhibit the activation of microglia and the protein expressions of inflammatory factors in wild‐type mice. However, in NLRP3 knockout mice, the microglia activation and high expression of inflammatory factors were not reduced after H‐k *L. m* treatment (Figure 8d).

## 4 DISCUSSION

The purpose of this study was to observe whether *L. m* conferred DA neuroprotection against PD. Results clearly showed that H‐k *L. m* protected DA neurons from 6‐OHDA‐induced DA neuronal loss but live *L. m* had no protective effects. Moreover, H‐k *L. m* could inhibit microglia activation and release of inflammatory factors. In addition, the DA neuroprotective effects of H‐k *L. m* disappeared in NLRP3 knockout mice. Together, this study provided the first evidence that H‐k *L. m* was beneficial for PD treatment.

A large number of studies confirmed that the humble intestinal flora harmonized the health of the host. Also, intestinal flora was affected by genetic, nutritional, and environmental factors.^35^ In the present study, high‐throughput sequencing was first performed on feces of three common PD animal models to explore the changes of intestinal flora. Then, this study found that *L. m* changed apparently in the 6‐OHDA model. *L. m* is the potential probiotic with the most beneficial effects. At present, there are few studies on *L. m* although current studies indicated that it had anti‐inflammatory effects.

This study demonstrated that 6‐OHDA could change the abundance and diversity of intestinal flora in rats, while no significant difference was exhibited in LPS‐ and ROT‐induced PD rat models. These findings were not fully consistent with the existed studies. For example, high‐throughput 16S rRNA gene sequencing revealed that gut microbial environment was apparently changed in 6‐OHDA‐induced mouse PD model.^36^ However, the mechanisms underlying how 6‐OHDA caused gut microbial environment alterations were unknown. On the other hand, previous studies showed that chronic infusion of ROT for 22–28 days induced enteric nervous system dysfunction and delayed gastric emptying, which might further affect the composition of gut microbiota.^37^, ^38^ Another evidence indicated that a bilateral intra‐nigral administration of LPS caused gastric dysmotility to further influence gut microbiota, which was consistent with human PD.^39^ Gastric dysmotility was closely associated with an imbalance of neurotransmitters in the dorsal motor nucleus of the vagus nerve. Despite understanding the direct role of LPS, ROT, and 6‐OHDA in PD pathophysiology, the mechanisms responsible for the early gastrointestinal tract complications associated with PD etiology was far from being elucidated.^40^ Here, there are several limitations in this study. First, the time‐course study on gut microbial changes would provide more detail information. Second, how 6‐OHDA affected gut microbial environment in rats was unrevealed. Thus, the mechanisms underlying 6‐OHDA‐induced alteration of microbes warrant further exploration. This study provides a baseline for understanding the microbial communities in 6‐OHDA‐induced PD rat model to further investigate the role of gut microbiota in PD pathogenesis.

At the beginning, the initial design of this study was mainly focused on live *L. m*, in which H‐k *L. m* was used as a negative control. According to the current publications, the administration time of probiotics was different, including 2 weeks (probiotics treatment for 1 week previous PD and Alzheimer's disease animal model establishment and followed for further 1 week)^41^ and 5 weeks (probiotics treatment for 2 weeks previous PD animal model establishment and followed for further 3 weeks).^42^ Considering the premise that live probiotics should spend more time to colonize in the intestinal tract, prophylactic treatment was applied to give live *L. m*. However, it was unexpectedly found that H‐k *L. m* produced neuroprotection against DA neuronal loss, whereas no significant neuroprotection was discerned after live *L. m* treatment. Although the underlying mechanisms remained unilluminated, it was quite interesting to investigate the reasons why H‐k *L. m* rather than live *L. m* produced neuroprotection. In addition, this study was a pilot study just demonstrating pre‐treatment of H‐k *L. m* could confer neuroprotection against DA neuronal loss. Next, to investigate whether post‐treatment of live or H‐k *L. m* exerted DA neuroprotection was an interesting area needs to be studied in future.

At present, the pathogenesis of PD is still unclear. However, the role of neuroinflammation in the occurrence and development of PD has been well confirmed. Microglia are brain resident immune cells, although they can promote neurogenesis and synaptic pruning by clearing debris to maintain brain development and the role of the steady state. In order to response from acute inflammatory cytokines and the stimulation of nerve toxicity, microglia are activated and produce various cytokines and chemokines.^43^ At the same time, DA neuronal damage and loss occurred after the abnormal activation of microglia.^44^ Therefore, inhibition of microglial activation might become a breakthrough in rescuing the degeneration and death of DA neurons. In the present study, we found that live *L. m* did not have neuroprotective effects on 6‐OHDA‐induced neuronal damage, while H‐k *L. m* showed a beneficial effect. Since current studies on bacteria is not limited to live bacteria, inactivated bacteria may also have excellent intestinal barrier protection, immunological regulation and anti‐inflammatory effects,^26^, ^45^, ^46^ which encouraged us to shift the research aim and concentrate it on H‐k *L. m*. In this study, H‐k *L. m* could protect DA neurons against 6‐OHDA‐induced neurotoxicity and inhibit microglia‐mediated neuroinflammation.

Cytokines secreted by activated microglia play an amplified role in the development of neuroinflammation. Moreover, a variety of pattern recognition receptors (PRRs) exist in microglia. Nucleotide‐binding oligomerization domain‐like receptor (NLR) is a PRR that could be activated by many endogenous or exogenous factors and usually acts as a sensor molecule for inflammatory activation.^47^ NLRP3 inflammasome activation is closely related to neurodegenerative diseases, such as PD.^48^ Various stimuli, such as ATP, bacterial toxins, and pathogen‐associated RNA, as well as certain cellular signals, such as K^+^ efflux, Ca^2+^ mobilization, Na^+^ influx and Cl^−^ efflux, ROS and mitochondrial dysfunction, affect NLRP3 inflammasome activation.^49^ Studies have shown that in PD animal models, different neurotoxins, such as LPS, ROT, 6‐OHDA, and 1‐methyl‐4‐phenyl‐1,2,3,6‐tetrahydropyridine (MPTP) were able to activate NLRP3 inflammasome by direct or indirect means, which initiated the inflammatory cascade and thus caused a continuous inflammatory environment, and ultimately damaged DA neurons.^50^, ^51^ However, inhibition of NLRP3 inflammasome activation increased the survival rate of DA neurons.^52^, ^53^ In the present study, activation of NLRP3 inflammasome was inhibited by H‐k *L. m*. Meanwhile, H‐k *L. m* decreased the production of IL‐1β and IL‐18. To further observe the role of NLRP3 as a neuroprotective target of H‐k *L. m*, NLRP3 knockout mice were applied. Results showed that H‐k *L. m* still protected DA neurons in wild‐type mice, while the protective effects of H‐k *L. m* on DA neurons disappeared in NLRP3 knockout mice. At the same time, microglia activation and increased inflammatory factors were not reduced in NLRP3 knockout mice, either. It was noted that the protein expressions of IL‐1β and IL‐18 were upregulated in 6‐OHDA and 6‐OHDA+H‐k *L. m* groups of NLRP3 knockout mice. It was because NLRP3 inflammasome signaling was actually involved in the production of IL‐1β and IL‐18 but NLRP3 inflammasome was not the only one pathway to regulate IL‐1β and IL‐18 generation and other signaling pathways, such as MAPK and NF‐κB signaling, also participated in IL‐1β and IL‐18 production during neuroinflammatory responses. In addition, this point was also supported by another phenomenon that if NLRP3 inflammasome was the only one pathway for IL‐1β and IL‐18 generation, 6‐OHDA was not capable to induce IL‐1β and IL‐18 production. However, 6‐OHDA still caused the upregulation of IL‐1β and IL‐18 protein expressions and H‐k *L. m* did not inhibit 6‐OHDA‐induced IL‐1β and IL‐18 expression upregulation. These data indicated that H‐k *L. m* attenuated 6‐OHDA‐induced DA neurotoxicity via the inhibition of microglial NLRP3 inflammasome activation. Next, to probe how H‐k *L. m* interfered with NLRP3 inflammasome and how NLRP3 knockout bypassed H‐k *L. m*‐mediated DA neuroprotection was an interesting area needed to be elucidated in future.

In the present study, live *L. m* did not present beneficial effects. According to the International Society for Probiotic and Probiotic Science, the premise of probiotic action is adequate administration.^54^ Unfortunately, although the administration dose of this experiment is already in the category of conventional administration dose of probiotics, due to the limitations of manual operation in the laboratory, the administration dose cannot be achieved. In addition, the colonization cycle of live probiotics may also be one of the reasons why they cannot act as soon as possible compared with the inactivated bacterial microbial components directly exposed to the intestine. In this study, heat treatment resulted in the loss of biological activity of lactic acid bacteria, thus reducing the potential risk of certain viable bacteria, such as disrupted bacterial balance, disrupted risk of translocation, and bacteremia and sepsis due to translocation, especially in immunocompromised, critically ill patients and pediatric populations.^55^, ^56^ Heat‐killed bacteria can exert probiotic properties under conditions that avoid the above risks. Indeed, bacterial viability or bacterial cell wall integrity is not a necessary condition for probiotics to exhibit beneficial effects. Microbial components of heat‐killed bacteria, such as extracellular polysaccharides, teichoic, and lipoteichoic acids, peptidoglycan are active at signal transduction receptors in intestinal epithelium, dendritic cells, and other immune enterocytes, stimulating the innate immune system, adaptive responses, and immunomodulatory activities.^57^ Therefore, although heat‐killed bacteria lose their biological viability compared with live bacteria, heat‐killed bacteria may still produce microbiologically similar effects in a non‐microbiological manner. For example, heat‐killed *Lactobacillus fermentum* and *Lactobacillus delbrueckii* could remodel mouse intestinal bacteria and affect the level of endocrine substance corticosterone.^58^ Moreover, heat‐killed *Lactobacillus brevis* SBC8803 enhanced mouse intestinal barrier^45^ and heat‐killed *Lactobacillus plantarum* KCTC13314BP and heat‐killed *Lactobacillus brevis* could increase the phagocytic activity of macrophages and produce immune stimulation by activating MAPK and STAT3 or TAK1 pathways.^59^, ^60^ In addition, heat‐killed lactic acid bacteria can transform T helper (Th) type 2 response to Th1 response and have immunoregulatory ability.^61^, ^62^ These beneficial effects exerted by heat‐killed bacteria were not different from those generated by live probiotics. Therefore, the immunoregulatory effects of live or dead probiotics might play an important role in the communication among gut microbiota, neuroendocrine system and brain.^63^, ^64^ It has been confirmed that live probiotics could influence the gastrointestinal microbiota and have immunomodulatory effects, while dead probiotics might generate anti‐inflammatory responses.^65^, ^66^ In this study, H‐k *L. m* presented neuroprotection against DA neuronal loss, whereas no DA neuroprotection was exhibited after live *L. m* treatment. This phenomenon was unusually speculated in several ways: (i) in contrary to live *L. m*, a new paradigm was created that H‐k *L. m* could also produce beneficial properties; (ii) H‐k *L. m* might eliminate the risk of leakage of other live bacteria systemically to further exert neuroprotection in brain; and (iii) several kinds of active components of H‐k *L. m* could influence the communication between gut microbiota and brain. However, the underlying mechanisms remains unclear and warrant further systematic investigation. Collectively, although the relative importance of live or dead probiotics‐mediated beneficial effects is difficult to evaluate, the improved safety, longer shelf‐life and relatively easy standardization are attractive advantages of dead probiotic preparations over live ones.

Until now, PD is based on drug therapy, which actually does not truly curb the degeneration of DA neurons, and long‐term use of these drugs is also accompanied by serious side effects.^67^ Thus, it is important to develop safe and effective potential drugs for the clinical treatment of PD. Studies have shown that neuroinflammation promotes the loss of DA neurons in the substantia nigra, and NLRP3 inflammasome contributes positively to neuroinflammation formation. Therefore, inhibition of NLRP3 inflammasome is considered to be an effective target for PD therapy. In this study, heat‐killed gut bacteria H‐k *L. m* was confirmed to have DA neuroprotective properties through the inhibition of NLRP3 inflammasome activation in microglia. These findings indicated the possibility that heat‐killed gut bacteria were involved in the regulation of neuroinflammation in the brain and further DA neuroprotection. In the future, whether H‐k *L. m* is effective in humans warrant further investigation in clinical studies.

## 5 CONCLUSIONS

This study demonstrated that H‐k *L. m* alleviated 6‐OHDA‐induced DA neurotoxicity via the inhibition of NLRP3 inflammasome activation in microglia.

## AUTHOR CONTRIBUTIONS

**Hong‐Xia Fan:** Data curation (lead); investigation (lead); methodology (equal); writing – original draft (lead). **Shuo Sheng:** Conceptualization (equal); data curation (equal); investigation (supporting); methodology (supporting). **Dai‐Di Li:** Data curation (equal); investigation (supporting); methodology (supporting). **Jing‐Jie Li:** Data curation (supporting); methodology (supporting). **Guo‐Qing Wang:** Data curation (supporting); methodology (supporting). **Feng Zhang:** Conceptualization (lead); data curation (lead); funding acquisition (lead); investigation (supporting); methodology (supporting); project administration (lead); writing – review and editing (lead).

## CONFLICT OF INTEREST

The authors declare that they have no any conflict of interests.

### PEER REVIEW

The peer review history for this article is available at https://publons.com/publon/10.1002/btm2.10455.

## Data Availability

The data and materials used in this manuscript were available from the corresponding author on reasonable request.
